# The uric acid/HDL-C ratio may predict significant coronary stenosis in moderate left main coronary artery lesions: an intravascular ultrasonography study

**DOI:** 10.1186/s12944-024-02193-y

**Published:** 2024-07-30

**Authors:** Ömer Furkan Demir, Abdulsamet Arslan, Mustafa Kınık, Barış Şensoy, Günseli Demir

**Affiliations:** 1 Department of Cardiology, Bursa Yuksek Ihtisas Training and Research Hospital, Bursa, Turkey; 2 Department of Cardiology, Bursa İnegöl State Hospital, Bursa, Turkey; 3 Department of Internal Medicine, Bursa Yuksek Ihtisas Training and Research Hospital, Bursa, Turkey

**Keywords:** Intravascular ultrasound, Coronary artery disease, Left main coronary artery, Uric acid, High-density lipoprotein cholesterol

## Abstract

**Background:**

There may be severe difficulties in determining the severity of LMCA (left main coronary artery) lesions. The use of intravascular ultrasound (IVUS) facilitates decisions about lesion severity in these patients. The aim of this study was to investigate the relationship between the UHR (uric acid to HDL-C ratio) and lesion severity in patients who underwent LMCA IVUS.

**Methods:**

This study included 205 patients with ICS (intermediate coronary stenosis) in the LMCA who underwent IVUS. In the IVUS measurements of these patients, the plaque burden (PB) and the minimal lumen area (MLA) showing lesion severity were measured.

**Results:**

The patients were separated into two groups according to plaque burden (< 65% and ≥ 65%). The UHR was significantly greater in the high plaque burden group (479.5 vs. 428.6, *P* = 0.001). When the patients were separated into two groups according to the MLA (< 6mm^2^ and ≥ 6mm^2^), the UHR was determined to be significantly greater in the group with low MLA (476.8 vs. 414.9, *P* < 0.001). In the ROC analysis performed according to the MLA and plaque burden values, the UHR cutoff value of 450 was found to have similar sensitivity and the same specificity for both parameters.

**Conclusions:**

The results of this study suggested that there is a relationship between UHR and MLA < 6mm^2^ and plaque burden ≥ 65%, which are independently evaluated as critical in IVUS, and this could predict anatomically significant lesions in patients with a moderate degree of LMCA stricture.

**Supplementary Information:**

The online version contains supplementary material available at 10.1186/s12944-024-02193-y.

## Introduction

Coronary artery disease (CAD) has been determined to be the leading cause of death in most countries worldwide. Coronary angiography (CAG) is one of the most commonly used methods to evaluate the extent and severity of CAD in clinical practice. However, the interpretation of CAG images can be prevented because vessels appear short or overlap on the image, and reliable evaluation of stenosis severity in advanced-stage eccentric lesions can be difficult [1]. When these challenges in the correct evaluation of strictures on angiography are taken into consideration, patients are usually classified as having intermediate coronary stenosis (ICS). An ICS is defined as angiographically detected stenosis between 30% and 70%. This presents physicians with significant clinical difficulty in terms of optimal lesion evaluation and management strategies for patients [2]. Although the prevalence of ICS in the general population is not well known, recorded data suggest that ICS could be present in up to 25% of patients evaluated by CAG [3]. In addition, left main coronary artery (LMCA) lesions are found in 5–7% of CAG patients [4], and determining the severity of LMCA lesions is highly difficult. The use of intravascular ultrasound (IVUS) facilitates the determination of lesion severity in these patients. However, IVUS cannot be widely used because of the need for extra time for the intervention, the need for an experienced operator, and the extra cost.

Some previous studies have shown that some biomarkers show consistency in lesion severity evaluation with tests that physiologically evaluate the severity of coronary lesions [5, 6]. It is also known that uric acid (UA) and high-density lipoprotein cholesterol (HDL-C) disorders are both risk factors for the development of CAD [7, 8]. Previous studies have evaluated the ratio of these two parameters to each other and their relationship with several diseases. In those studies, the uric acid-to-HDL-C ratio (UHR) was shown to be related to the development of hypertension [9], hepatic steatosis [10], and cardiovascular mortality [11]. According to a recent study in the literature, compared with the UHR and HDL-C parameters alone, the UHR is a successful predictor of functionally critical lesions and a better predictor of critical lesions [12]. The ratio of serum UHR, which has proven cardiovascular effects, may be a good biomarker of CAD severity and the severity of the lesion in patients with ICS detected on angiography.

The primary aim of this study was to investigate the relationship between UHR and lesion severity in patients who underwent LMCA IVUS. Thus, in patients with ICS detected in the LMCA and for whom IVUS was performed, the UHR parameter, which can be easily measured before the IVUS procedure, can predict the severity of the lesion and prevent unnecessary IVUS procedures. There is no previous study in the literature that has investigated the relationship between UHR and lesion severity on LMCA IVUS.

## Materials and methods

### Data collection and laboratory analysis

In this retrospective, planned, observational study, patients who underwent CAG at a top-level cardiac center and were diagnosed with CAD, were diagnosed with ICS in the LMCA as a result of CAG, and therefore underwent IVUS measurement. Non-ST segment elevation myocardial infarction (NSTEMI) and unstable angina pectoris (USAP) patients included in the study were defined as having acute coronary syndrome (ACS), and other patients were defined as having stable angina pectoris (SAP). The patients included were those without complications (no ulceration, dissection, or thrombus), who were diagnosed with ICS and unprotected LMCA lesions. All patients treated between January 2020 and June 2023 were included in the study.

The inclusion criteria for this study were (i) age in the range of 20–85 years, (ii) indication for CAG with a diagnosis of SAP or ACS, and (iii) lesion determined on the LMCA as a result of CAG. Exclusion criteria from this study were (i) cardiogenic shock, (ii) severe heart valve disease, (iii) active malignancy or active infection, and (iv) incomplete clinical data for uric acid or HDL-C.

A total of 205 patients who met the inclusion criteria were included in this study. The patient population was classified according to the IVUS measurements by evaluating 2 criteria. The patients were divided into 2 groups according to the healthy vessel diameter measured on IVUS, minimal lumen area (MLA) < 6mm^2^ and ≥6mm^2^, and the plaque burden (PB) measured on IVUS was < 65% and ≥ 65%, respectively. The limits defined for MLA and PB were determined according to the limits determined for IVUS in previous studies [13, 14]. During the study, all the patients were treated in the hospital according to the current guidelines. IVUS was not performed on patients who presented with ACS in the acute period. After the patients stabilized, IVUS was performed. The basic demographic characteristics of the patients and their comorbidities, such as hypertension, diabetes mellitus, and hyperlipidemia, together with laboratory parameters and angiographic findings, were retrieved from the hospital database. From a scan of the archived records, data were also obtained for all the patients with previous ECG recordings and previous drug use.

Routine blood tests of the patients at the time of admission were performed via the hospital system, and hemoglobin levels, white blood cell counts, platelet counts and biochemical data were recorded. Total cholesterol, high-density lipoprotein cholesterol and low-density lipoprotein cholesterol values were calculated from blood tests taken after an 8–12 h overnight fast. The eGFR was calculated according to the MDRD formula (eGFR = 186 x [creatinine/88.4]-1.154 x [age]− 0.203 x [0.742 if female, 1.210 if black]) [14].

### Coronary angiography

Standard coronary angiography was performed using a 5 or 6 French Judkins diagnostic catheter (Boston Scientific, MA, USA) and a femoral or radial approach. The reference vessel diameter was taken as the average of angiographically normal segments 10 mm in length proximal and distal to the lesion. When a normal, clear segment could not be identified proximally (e.g., location of the ostial lesion), only the distal segment was included in the analysis.

### IVUS Imaging and analysis

The operators were not blinded to the angiography images. IVUS imaging was performed after intracoronary nitroglycerine administration. The examinations were conducted using a system produced by Cardiovascular Imaging Systems/Boston Scientific Corporation. The IVUS catheter was advanced approximately 10 mm distal to the lesion, video recording was started, and the coronary artery was imaged retrogradely up to the aorta. IVUS images were recorded by retracting at a speed of 0.5 mm/s using a motorized pull-back system. The examinations were recorded on the integral memory of the Boston device for offline analysis.

Normal coronary anatomy, plaque composition of coronary vessels, and measurements calculated using IVUS have been reported [15, 16]. The external elastic membrane (EEM) cross-sectional area was measured by following the anterior edge of the vascular adventitia. The lesion region is the cross-sectional slice with the smallest lumen, and from the sections with the same lumen area, the section that included the highest plaque burden was selected. If the plaque around the catheter was compressed, the vessel lumen was assumed to be the physical size (not acoustic) of the catheter. For the reference segment, the average was taken of the most normal visual slices (the largest lumen containing the least plaque) from among the main branches 5 mm proximal and distal to the lesion. For ostial lesions, the distal reference was used. IVUS measurements of the patients were recorded, and the IVUS measurements were performed according to the American Cardiology College and European Cardiology Society standards [17].

Manually taken recordings were examined, and the following lesion and reference measurements were performed in diastole: EEM cross-sectional areas (CSA), lumen CSA, MLA and PB ([vessel area – lumen area]/vessel area).

### Statistical analysis

All the statistical analyses were performed using SPSS vn. 20 software (Statistical Package for the Social Sciences). The normality of the data was assessed using the Kolmogorov–Smirnov or Shapiro–Wilk test. Variables are reported as the mean ± standard deviation (SD) or median and interquartile range. According to the normality test results, the independent samples t test or the Mann‒Whitney U test was used for between-group comparisons. For the analysis of categorical data, a χ2 test or Fisher’s exact test was used if any expected cell count was < 5, and descriptive statistics are presented as numbers (n) and percentages (%). To evaluate the correlations between the UHR and anatomically significant atherosclerotic parameters (plaque burden and MLA), Spearman’s ρ correlation analysis was performed. ROC curve analysis was applied to determine the cutoff point and the area under the curve (AUC) of the UHR for significant anatomic atherosclerosis. The UHR was used as a prespecified dichotomous variable to facilitate meaningful clinical interpretation of the results. When indicated, analyses performed using UHR as a continuous variable were also reported. Univariate binary logistic regression analysis was used to investigate the relationship between significant anatomic atherosclerosis and UHR (separately for plaque burden and MLA). The effect of the UHR on atherosclerosis severity was reported using the odds ratio (OR) and 95% CI. A value of *P* < 0.05 was considered to indicate statistical significance, and the statistical tests were two-sided.

### Ethics approval and consent to participate

All the procedures in this study, including human participants, were applied in compliance with the ethical standards of the institutional research committee and the 1964 Helsinki Declaration and subsequent revisions or comparable ethical standards. No animals were used in this study. Approval for the study was granted by the Bursa Yuksek Ihtisas Training and Research Hospital Ethics Committee (Protocol Code: 2011-KAEK-25 2023/11 − 02).

## Results

The present study included 205 patients who underwent CAG with a diagnosis of ACS or SAP and who were diagnosed with an LMCA lesion on CAG and underwent IVUS. The patients comprised 156 (76.1%) males and 49 (23.9%) females with a median (IQR) age of 61 (53–68) years. CAG was performed because of a diagnosis of ACS in 114 (55.6%) patients and SAP in 91 (44.4%). According to the IVUS results, the mean MLA was 6.4 (4.8–9.1) mm^2^, and the percentage of plaque burden was 58% (46–67%).

The demographic, clinical and laboratory data of the patients were compared between the two groups according to whether the percentage of PB in the LMCA IVUS was < 65% or ≥ 65% (Table 1). No significant difference was detected between these two groups with respect to demographic characteristics or comorbidities. When the patients were compared in terms of medication use, B-blocker use (*P* = 0.002) and ACE inhibitor use (*P* = 0.022) were significantly more common in the PB < 65% group. Additionally, compared with uric acid-lowering drugs, no significant difference was found between the groups in terms of allopurinol use. The use of other uric acid-lowering drugs, such as probenecid and febuxostat, was not detected in our patients. When laboratory parameters were examined, it was determined that the uric acid level was significantly greater in the group with high PB (*P* = 0.002). When the continuous variable of the UHR parameter, which shows the relationship between uric acid and HDL-C, was examined, it was determined that it was significantly greater in the group with high PB (479.5 vs. 428.6, *P* = 0.001).

**Table 1:** The baseline characteristics and laboratory data of all patients according to LMCA plaque burden

		All patients (*n* = 205)	Plaque burden ≥%65 (*n* = 74)	Plaque burden < 65 (*n* = 131)	*p* value
**Demographic characteristics**					
Age, y		61 (53–68)	60 (55–71)	61 (53–68)	0.186
Male sex, %		156 (76.1)	55 (74.3)	101 (77.1)	0.734
Body mass index, kg/m^2^		26.8 (24.7–29)	26.2 (24.5–29.3)	26.8 (24.8–28.9)	0.605
**Comorbidites**					
Hypertension, %		129 (62.9)	45 (60.8)	84 (64.1)	0.654
Diabetes mellitus, %		48 (23.4)	19 (25.7)	29 (22.1)	0.608
Hyperlipidemia, %		60 (29.3)	27 (36.5)	33 (25.2)	0.110
Smoking, %		132 (64.4)	47 (63.5)	85 (64.9)	0.880
Chronic kidney disease, %		31 (15.1)	12 (16.2)	19 (14.5)	0.840
**Medications**					
Acetylsalicylic Acid Use, %		204 (99.5)	74 (100)	130 (99.2)	1.000
P2Y12 Inh Use, %	Clopidogrel	103 (50.2)	40 (54.1)	63 (48.1)	0.468
	Ticagrelor	44 (21.5)	22 (29.7)	22 (16.8)	
	Prasugrel	16 (7.8)	6 (8.1)	10 (7.6)	
B-Blocker Use, %		191 (93.2)	60 (82.4)	131 (100)	0.002
ACE Inh Use, %		175 (85.4)	52(81.1)	123(93.9)	0.022
Statin Use, %		200 (97.6)	73 (98.6)	127 (96.9)	0.656
OAD Use, %		50 (24.4)	24 (32.4)	26 (19.8)	0.062
Allopurinol Use, %		16 (7.8)	6 (8.1)	10 (7.6)	0.716
**Clinical Presentation**					
Acute Coronary Syndrome, %		114 (55.6)	47 (63.5)	67 (51.1)	0.094
Stable Angina Pectoris, %		91 (44.4)	27 (36.5)	64 (48.9)	
**Laboratory assessment**					
Hemoglobin, g/dl		13.3 (12.1–14.5)	12.8 (11.7–14.3)	13.6 (12.2–14.6)	0.085
eGFR, ml/min/1.73 m^2^		90.5 (70-101.6)	92.8 (70-101.5)	90.1 (69.8-102.1)	0.904
Total cholesterol, mmol/L		4.37 (3.74–5.3)	4.25 (3.43–5.30)	4.52 (3.87–5.36)	0.086
Triglycerides, mmol/L		1.56 (1.01–2.41)	1.41 (0.97–2.16)	1.60 (1.06–2.53)	0.279
LDL cholesterol, mmol/L		2.39 (1.78–3.33)	2.31 (1.57–3.07)	2.44 (1.84–3.48)	0.123
HDL cholesterol, mmol/L		1.07 (0.94–1.25)	1.07 (0.91–1.25)	1.08 (0.94–1.26)	0.456
Albumin, g/L		42.1 (39.1–44.5)	41.7 (38.4–44.3)	42.1 (39.1–44.6)	0.115
HbA1c, %		5.78 (5.5–6.4)	5.89 (5.6–6.5)	5.69 (5.4–6.4)	0.430
Uric asid, µmol/L		477.4 (415.5-548.1)	503.9 (459.7-565.8)	468.5 (406.6-539.2)	0.002
UHR, continuous variable		446.3 (366-518.1)	479.5 (400.4–570)	428.6 (357-494.3)	0.001
UHR ≤ 450, categorical variable		109 (53,2)	28 (37.8)	81 (61.8)	0.001
IVUS LMCA MLA, mm^2^		6.4 (4.8–9.1)	4.9 (3.5–5.8)	8.7 (6.4–10.9)	< 0.001
IVUS LMCA plaque burden, %		58 (46–67)	69 (66–72)	48 (41–56)	< 0.001

The data are presented as the median (interquartile range) or number (percentage) of patients. Abbreviations: ACE, angiotensin converting enzyme; eGFR, estimated glomerular filtration rate; HDL, high-density lipoprotein; IQR, interquartile range; IVUS, intravascular ultrasound; LDL, low-density lipoprotein; LMCA, left main coronary artery; MLA, minimal lumen area; OAD, oral antidiabetic; UHR, UA-to-HDL-C ratio

**Fig. 1:**
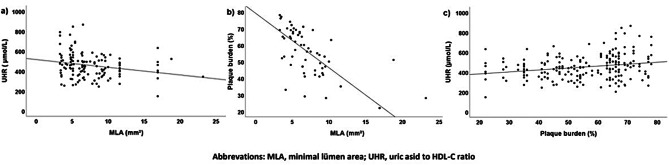
Correlations between UHR and atherosclerotic severity variables; (**a**) correlations between UHR and MLA, (**b**) correlations between MLA and plaque burden, (**c**) correlations between plaque burden and UHR

The correlations between the severity of anatomic atherosclerosis and the UHR were evaluated using Spearman’s ρ correlation analysis. Both the plaque burden and MLA were mildly correlated with the UHR. MLA was negatively associated with the UHR, and plaque burden was positively correlated with the UHR (rs = -0.26, *P* = 0.002; rs = 0.22, *P* = 0.001, respectively) (Fig. 1).

**Fig. 2:**
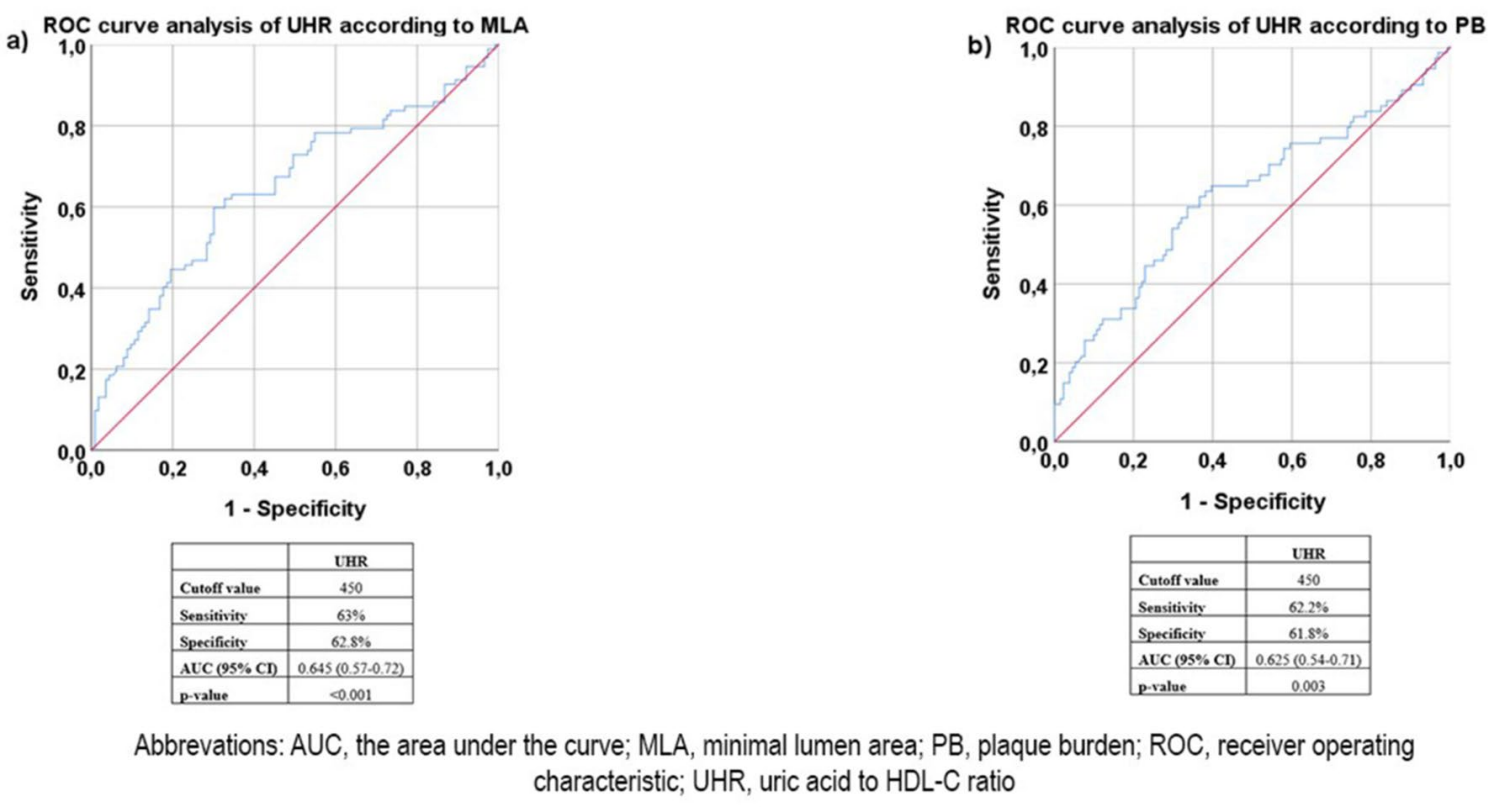
ROC curve analysis of the UHR according to minimal lumen area and ROC curve analysis of the UHR according to plaque burden

The results of the receiver operating characteristic (ROC) analysis performed according to the MLA and PB values of the patients are shown in Fig. 2. When the ROC analyses were applied separately to the IVUS parameters of MLA and PB with UHR, the UHR cutoff value of 450 for both parameters of anatomic atherosclerosis was determined to have similar sensitivity and the same specificity.

When the UHR was examined as a categorical variable, it was determined that the number of patients with a UHR ≤ 450 was significantly greater in the low-PB group (81 patients vs. 28 patients, *P* = 0.001). No significant differences were detected in the other laboratory parameters. Multivariate analysis of the parameters UHR and ACE inhibitor efficacy on PB revealed that the UHR was an independent predictor of high PB (OR = 1.004, 95% CI: 1.001–1.007).

The demographic, clinical and laboratory data of the patients were compared between those with an MLA < 6mm^2^ and those with an MLA ≥ 6 mm^2^ according to the LMCA IVUS measurements. (Table 2). No significant difference was found between the 2 patient groups in terms of demographic characteristics or comorbidities. When the patients were compared in terms of medication use, B-blocker use (*P* = 0.004) and ACE inhibitor use (*P* = 0.003) were found to be significantly more common in the patient group with MLA ≥ 6mm^2^. No significant difference was found between the groups in terms of uric acid-lowering medications. When the laboratory parameters were examined, the UHR was determined to be significantly greater in the low MLA group (476.8 vs. 414.9, *P* < 0.001). The UHR was examined as a categorical variable, and it was determined that the number of patients with a UHR ≤ 450 was significantly greater in the high MLA group (74 patients vs. 35 patients, *P* < 0.001). The PB percentage was significantly greater in the low MLA group (*P* < 0.001). Multivariate analysis of the parameters UHR and ACE inhibitor efficacy on MLA revealed that the UHR was an independent predictor of low MLA (OR = 0.996, 95% CI: 0.993–0.998). Although the uric acid value seems to be an effective parameter for MLA and PB, the uric acid parameter was not included in multivariate analyses due to the multicollinearity between uric acid and UHR parameters. Additionally, due to the strong relationship between the use of ace inhibitors and b-blockers, multivariate analyses were performed using only ace inhibitors and UHR parameters.

**Table 2:** The baseline characteristics and laboratory findings of all patients according to LMCA minimum lumen area

		All patients (*n* = 205)	MLA < 6 mm^2^ (*n* = 92)	MLA ≥ 6 mm^2^ (*n* = 113)	*p* value
**Demographic characteristics**					
Age, y		61 (53–68)	60 (55–70)	61 (52–68)	0.158
Male sex, %		156 (76.1)	72 (78.3)	84 (74.3)	0.512
Body mass index, kg/m^2^		26.8 (24.7–29)	26 (24.2–28.6)	27 (24.9–29)	0.097
**Comorbidites**					
Hypertension, %		129 (62.9)	58 (63)	71 (62.8)	1.000
Diabetes mellitus, %		48 (23.4)	25 (27.2)	23 (20.4)	0.320
Hyperlipidemia, %		60 (29.3)	31 (33.7)	29 (25.7)	0.221
Smoking, %		132 (64.4)	56 (60.9)	76 (67.3)	0.380
Chronic kidney disease, %		31 (15.1)	14 (15.2)	17 (15)	1.000
**Medications**					
Acetylsalicylic Acid Use, %		204 (99.5)	92 (100)	112 (99.1)	1.000
P2Y12 Inh Use, %	Clopidogrel	103 (50.2)	50 (54.3)	53 (46.9)	0.327
	Ticagrelor	44 (21.5)	27 (29.3)	17 (15)	
	Prasugrel	16 (7.8)	7 (7.6)	9 (8)	
B-Blocker Use, %		191 (93.2)	81 (85.9)	110 (97.3)	0.004
ACE Inh Use, %		175 (85.4)	70 (79.3)	105 (93)	0.003
Statin Use, %		200 (97.6)	91 (98.9)	109 (96.5)	0.382
OAD Use, %		50 (24.4)	27 (29.3)	23 (20.4)	0.145
Allopurinol Use, %		16 (7.8)	7 (7.6)	9 (8)	0.738
**Clinical Presentation**					
Acute Coronary Syndrome, %		114 (55.6)	60 (65.2)	54 (47.8)	0.074
Stable Angina Pectoris, %		91 (44.4)	32 (34.8)	59 (52.2)	
**Laboratory assessment**					
Hemoglobin, g/dl		13.3 (12.1–14.5)	13.1 (11.9–14.4)	13.6 (12.2–14.5)	0.630
eGFR, ml/min/1.73 m^2^		90.5 (70-101.6)	92.5 (70-101.5)	88.5 (70-101.6)	0.995
Total cholesterol, mmol/L		4.37 (3.74–5.3)	4.26 (3.44–5.31)	4.53 (3.88–5.37)	0.084
Triglycerides, mmol/L		1.56 (1.01–2.41)	1.41 (0.97–2.16)	1.62 (1.08–2.55)	0.259
LDL cholesterol, mmol/L		2.39 (1.78–3.33)	2.29 (1.55–3.08)	2.47 (1.84–3.51)	0.096
HDL cholesterol, mmol/L		1.07 (0.94–1.25)	1.06 (0.91–1.24)	1.08 (0.96–1.28)	0.107
HbA1c, %		5.78 (5.5–6.4)	5.88 (5.5–6.4)	5.69 (5.3–6.4)	0.353
Uric asid, µmol/L		477.4 (415.5-548.1)	503.9 (446.4-565.8)	468.5 (406.6-521.6)	0.012
UHR, continuous variable		446.3 (366-518.1)	476.8 (405.5–560)	414.9 (349.4–489)	< 0.001
UHR ≤ 450, categorical variable		109 (53.2)	35 (38)	74 (65.5)	< 0.001
IVUS LMCA MLA, mm^2^		6.4 (4.8–9.1)	4.8 (3.6–5.1)	8.9 (6.7–11.6)	< 0.001
IVUS LMCA plaque burden, %		58 (46–67)	66.5 (64–71)	47 (40–55)	< 0.001

The data are presented as the median (interquartile range) or number (percentage) of patients. Abbreviations: ACE, angiotensin converting enzyme; eGFR, estimated glomerular filtration rate; HDL, high-density lipoprotein; IQR, interquartile range; IVUS, intravascular ultrasound; LDL, low-density lipoprotein; LMCA, left main coronary artery; MLA, minimal lumen area; OAD, oral antidiabetic; UHR, UA-to-HDL-C ratio

The angiographic characteristics and IVUS parameters of the patients were compared according to UHR ≤ 450 and > 450 (Table 3). The number of critical vessels determined angiographically was significantly greater in the high UHR group (*P* = 0.039). When the CAG results were compared, the number of patients for whom CABG was chosen was significantly greater in the high-UHR group (*P* = 0.017). According to the IVUS parameters, the lumen volume was significantly lower in the group with a high UHR (*P* = 0.012). MLA values were determined to be significantly lower in the high UHR group (6.7 mm^2^ vs. 8.2 mm^2^, *P*: 0.030). When the MLA parameter was examined as a categorical variable, the number of patients with an MLA < 6mm^2^ was significantly greater in the high UHR group (57 patients vs. 35 patients, *P* < 0.001). The percentage of PB was determined to be significantly greater in the high UHR group (*P* = 0.002). When the PB percentage was examined as a categorical variable, the number of patients with a PB > 65% was significantly greater in the high UHR group (*P* = 0.001).

**Table 3:** Angiographic and procedural status of patients according to UHR

Angiographic parameters		All patients (*n* = 205)	UHR ≤ 450 (*n* = 109)	UHR > 450 (*n* = 96)	*p* value
**Procedural data**					
SYNTAX score		11 ± 10	10 ± 10	12 ± 9	0.061
Critical number of vessels, %	0	46 (22.4)	33 (30.3)	13 (13.5)	0.039
	1	64 (31.2)	33 (30.3)	31 (32.3)	
	2	59 (28.8)	26 (23.9)	33 (34.4)	
	3	35 (17.1)	16 (14.7)	19 (19.8)	
	4	1 (0.5)	1 (0.9)	0	
CAG result	Medical follow up	80 (39)	51 (46.8)	29 (30.2)	0.017
	PCI	73 (35.6)	38 (34.9)	35 (36.5)	
	CABG	52 (25.4)	20 (18.3)	32 (33.3)	
**IVUS-based volume parameters in LMCA**					
EEM volume, mm^3^		148.9 ± 47	151.3 ± 45.2	146.2 ± 49	0.434
Lumen volume, mm^3^		66.5 ± 35.7	72.4 ± 36.9	59.8 ± 33.4	0.012
Plaque volume, mm^3^		82.4 ± 32.1	78.9 ± 28.8	86.4 ± 35.1	0.095
MLA, mm^2^		7.4 ± 3.8	8.2 ± 3.9	6.7 ± 3.5	0.030
MLA < 6 mm^2^		92 (44.9)	35 (32.1)	57 (59.4)	< 0.001
Plaque burden, %		55.6 ± 14.5	52.7 ± 14.2	59 ± 14.2	0.002
Plaque burden ≥ 65%		74 (36.1)	28 (25.7)	46 (47.9)	0.001

The data are presented as the means ± SDs or numbers (percentages) of patients. Abbreviations: CABG, coronary artery bypass surgery; CAG, coronary angiography; EEM, external elastic membrane; IVUS, intravascular ultrasound; MLA, minimal lumen area; PCI, percutaneous coronary intervention; SD, standard deviation; SYNTAX, synergy between percutaneous coronary intervention with taxus and cardiac surgery; UHR, UA-to-HDL-C ratio

## Discussion

The results of this study demonstrated that significantly greater UHR values were detected in patients with a critical lesion according to the two separate parameters of MLA and plaque burden in the IVUS measurements of patients treated with an ICS on the LMCA. The UHR showed a negative correlation with the MLA, as determined by LMCA IVUS, and a positive correlation with the PB. The UHR, a novel biomarker combining UA and HDL-C, showed excellent diagnostic capacity for anatomically significant stenosis in patients with ICS detected in the LMCA. This is the first study in the literature to investigate the anatomical relationship between UHR and lesion severity in patients with ICSs detected in the LMCA.

Despite advances in the field of interventional cardiology, correct evaluation of ICS in the catheterization laboratory and the decision for PCI are still great challenges [18]. This problem has been resolved by determining the plaque burden and lesion severity with MLA limits through comparisons of IVUS measurements in LMCA lesions with distal and proximal vessel diameters. An MLA < 6mm^2^ and plaque burden ≥ 65% indicate anatomically critical LMCA stenosis [13, 19]. However, IVUS devices are not widely used for diagnosis and treatment in interventional cardiology in clinical practice due to the extra operating time, high cost, and the need for an experienced operator [20]. Therefore, there seems to be a need for a biomarker that is reliable in terms of showing lesion severity in ICS lesions. Several biomarkers have been investigated in previous studies to determine the severity of lesions in patients diagnosed with ICS. However, those studies have focused more on the functional significance of the stricture. Erdoğan M. et al. suggested that the systemic immune inflammation index, calculated as the number of neutrophils*platelets/lymphocytes, could predict fractional flow reserve (FFR) with high sensitivity and specificity [5]. Other studies have also shown that the UA value and modification of some lipid biomarkers could predict the FFR [6, 21]. In a recent study by Fanqi Li et al., a significant relationship was found between UHR and FFR severity in patients with ICSs [12]. All of these studies concentrated on the functional significance of the stricture and were conducted with the FFR test.

IVUS imaging is the current gold standard method for evaluating LMCA lesions [22]. In intermediate LMCA lesions, the FFR test often causes incorrect classification [23]. In addition, because of the high cost, reimbursement for IVUS imaging is only received for LMCA lesions in Turkey. Therefore, only patients with LMCA lesions were included in this study.

In the current study population, uric acid levels were determined to be significantly greater in patients with a high plaque burden. In a previous study by Ando K. et al. on this subject, a significant relationship was found between high uric acid and high lipid volume and plaque burden on IVUS [24]. Unlike the current study, that study included IVUS measurements for vessels other than the LMCA, and the effect of uric acid on plaque burden in particular was shown to be independent of sex. In this study, it was determined that uric acid levels were significantly greater in patient group with < 6mm^2^. As there was a relationship between uric acid levels and a high plaque burden, it was expected that the MLA would be low in patients with high uric acid levels.

When the UHR was examined as a continuous variable, a statistically significant relationship was determined between the UHR and high plaque burden and low MLA. The HDL-C parameter alone, independent of uric acid, was not found to have a significant effect on these measurements. This result could be associated with being lower in the group with high plaque burden and low MLA values, even if the HDL-C values were not significantly different. This can explain the significant effect of the HDL-C value on plaque burden and MLA compared to that of uric acid.

When ROC curve analyses were performed for MLA and plaque burden separately with the UHR parameter, the UHR cutoff value was found to be 450 for both curves. This demonstrated that a value of 450 can be defined as the limit for the UHR parameter. The patient population was then evaluated as subgroups above and below the UHR of 450. The number of patients with an MLA < 6mm^2^ and a plaque burden ≥ 65% was significantly greater in the group with a high UHR. Recently, in an FFR-related study, the UHR cutoff value was determined to be 310.8 [21]. This determined cutoff value was much lower than that in our current study, and this difference can be attributed to the measurement methods used. The abovementioned study evaluated the functionality of the stricture using an FFR device, whereas anatomic stricture evaluation was performed in the current study using IVUS.

In this study, the rate of B-blocker and ACE inhibitor use was significantly lower in the patient group with an MLA < 6 mm^2^ and the patient group with a plaque burden ≥ 65%. Previous studies in the literature have shown that B-blocker use slows the progression of coronary atherosclerosis [25]. It has also been shown that ACE inhibitor use significantly reduces coronary atherosclerosis [26]. Consistent with the findings of those studies, the use of B-blockers and ACE inhibitors was determined to be high in the current study in patients with low plaque burden and high MLA.

These results indicate that the UHR can predict vessel stricture, which is consistent with the findings of the MLA and plaque burden showing critical vascular narrowing on IVUS measurements in patients with ICS LMCA lesions. Thus, the UHR can predict MLA < 6mm^2^ and plaque burden ≥ 65%, which are considered critical for LMCA IVUS measurements.

### Study strengths and limitations

The strength of the current study lies in the fact that it is the first study to show the relationship between the anatomical severity of the lesion and UHR in patients who underwent IVUS due to residual LMCA lesions. The results obtained showed that there was a close relationship between UHR and the anatomical severity of the lesion in patients with intermediate LMCA lesions. A negative relationship was found between the MLA value measured in IVUS and UHR, and a positive relationship between PB and UHR. On the other hand, there were several limitations to this study. First of all, this study was designed as a single-center, retrospective study and had a limited sample size. Only patients with LMCA lesions were included in the study because IVUS is the gold standard method for LMCA lesions, and in Turkey, reimbursement for the use of the IVUS device is only made for LMCA lesions. A further limitation was that the rate of statin use was high in the patient population, which could have affected the HDL-C value in the UHR calculation. However, as there was no significant difference in the rate of statin use between those with and without critical lesions, statin use was not considered to have affected the results obtained. In the analyses, the AUCs ​​of the UHR and uric acid parameters were compared. Although the AUC for the UHR was greater, this difference was not statistically significant. However, since there are studies in the literature related to UHR and atherosclerosis incidence, the study was continued with this parameter.

## Conclusion

The results of this study suggested that there was a relationship between UHR and MLA < 6mm^2^ and plaque burden ≥ 65%, which are independently evaluated as critical in IVUS, and this could predict anatomically significant lesions in patients with a moderate degree of LMCA stricture. The UHR can be a surrogate marker for invasive treatment modalities for the management of patients with lesions of the LMCA in the gray zone.

## Electronic supplementary material

Below is the link to the electronic supplementary material.

Supplementary Material 1

Supplementary Material 2

## Data Availability

The datasets used and/or analysed during the current study are available from the corresponding author on reasonable request.

## Abbreviations

CAD: Coronary artery disease
CAG: Coronary angiography
ICS: Intermediate coronary stenosis
LMCA: Left main coronary artery
IVUS: Intravascular ultrasound
UA: Uric acid
HDL-C: High-density lipoprotein cholesterol
UHR: Uric acid to HDL-C ratio
SAP: Stable angina pectoris
ACS: Acute coronary syndrome
MLA: Minimal lumen area
PB: Plaque burden
EEM: External elastic membrane
CSA: Cross-sectional area
SD: Standard deviation
AUC: Area under the curve
OR: Odds ratio
