# Supplementary figures and images for: The uric acid/HDL-C ratio may predict significant coronary stenosis in moderate left main coronary artery lesions: an intravascular ultrasonography study
Source: Lipids Health Dis. 2024 Jul 30;23:233. doi: 10.1186/s12944-024-02193-y (PMC11289968; doi:10.1186/s12944-024-02193-y)

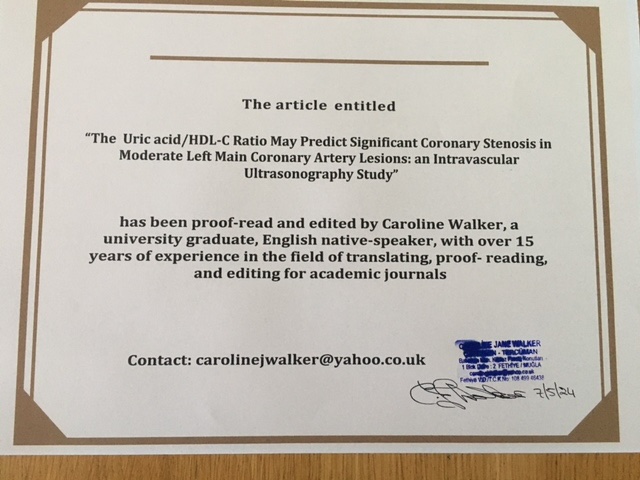

Supplement: Supplementary file 1 — Supplementary Material 1 [file 12944_2024_2193_MOESM1_ESM.jpg]
