# Supplementary material for: The uric acid/HDL-C ratio may predict significant coronary stenosis in moderate left main coronary artery lesions: an intravascular ultrasonography study
Source: Lipids Health Dis. 2024 Jul 30;23:233. doi: 10.1186/s12944-024-02193-y (PMC11289968; doi:10.1186/s12944-024-02193-y)
Supplement: Supplementary file 2 — Supplementary Material 2 [file 12944_2024_2193_MOESM2_ESM.pdf]

makalE

by Ismail Sivri

Submission date: 09-May-2024 06:30PM (UTC+0300)
Submission ID: 2375180296
File name: Turnitin_Similarity.docx (40.66K)
Word count: 5426
Character count: 29416

ABSTRACT

Background: There may be severe difficulties in determining the severity of LMCA (left main coronary artery) lesions. The use of intravascular ultrasound (IVUS) facilitates the decision about the lesion severity in these patients. The aim of this study was to investigate the relationship between UHR (uric acid to HDL-C ratio) and lesion severity in patients who have undergone LMCA IVUS.
Methods: The study included 205 patients who were determined with ICS (intermediate coronary stenosis) in LMCA and underwent an IVUS procedure. In the IVUS measurements of these patients, the plaque burden (PB) and the minimal lumen area (MLA) showing lesion severity were measured.
Results: The patients were separated into two groups according to the plaque burden values as those $<65\%$ and $\geq 65\%$. The UHR values were determined to be significantly higher in the high plaque burden group (479.5 vs. 428.6, p:0.001). When the patients were separated into two groups according to the MLA values ($<6mm^2$ and $\geq 6mm^2$), the UHR values were determined to be significantly higher in the group with low MLA values (476.8 vs. 414.9, $p<0.001$). In the ROC analysis performed according to the MLA and plaque burden values, the UHR cutoff value of 450 was found to have similar sensitivity and the same specificity for both parameters.
Conclusions: The results of this study suggested that there is a relationship between the UHR parameter and MLA $<6mm^2$ and plaque burden $\geq 65\%$, which are independently evaluated as critical in IVUS, and this could predict anatomically significant lesions in patients with a moderate degree of LMCA stricture.

Keywords: Intravascular ultrasound, coronary artery disease, left main coronary artery, uric acid, high-density lipoprotein cholesterol

The Uric acid/HDL-C Ratio May Predict Significant Coronary Stenosis in Moderate Left Main Coronary Artery Lesions: an Intravascular Ultrasonography Study

INTRODUCTION

Coronary artery disease (CAD) has been determined to be the leading cause of death in most countries around the world. Coronary angiography (CAG) is one of the most commonly used method to evaluate the extent and severity of CAD in clinical practice. However, the interpretation of CAG images can be prevented because of vessels appearing short or overlapping on the imaging, and there can be difficulties in the reliable evaluation of stenosis severity in advanced stage eccentric lesions [1]. When these challenges in the correct evaluation of stricture on angiography are taken into consideration, patients are usually entered in the category named 'intermediate coronary stenosis' (ICS). ICS is defined as angiographically detected stenosis between 30% and 70%. This presents physicians significant clinical difficulty in terms of optimal lesion evaluation and management strategy of patients [2]. Although the prevalence of the presence of ICS in the general population is not well known, recorded data suggest that ICS could be present in up to 25% of patients evaluated by CAG [3]. In addition, left main coronary artery (LMCA) lesions are determined in 5-7% of CAG patients [4] and there are grreat difficulties in determining the severity of LMCA lesions. The use of intravascular ultrasound (IVUS) facilitates the decision about the lesion severity in these patients. However, IVUS cannot be widely used because of the need for extra time for the intervention, an experienced operator, and extra cost.

Some previous studies have shown that some biomarkers show consistency in lesion severity evaluation with the tests that physiologically evalute the severity of coronary lesions [5, 6]. It is also known that uric acid (UA) and high-density lipoprotein cholesterol (HDL-C) disorders are both evaluated as risk factors for the development of CAD [7, 8]. Previous studies have evaluated the ratio of these two parameters with each other and the relationship with some diseases. In those studies, there has been shown to be a relationship of the uric acid to HDL-C ratio (UHR) with development of hypertension [9], hepatic steatosis [10], and cardiovascular mortality [11]. The ratio of serum UHR, which has proven cardiovascular effects, may be a good biomarker showing CAD severity and the severity of the lesion in patients with ICS detected on angiography.

The primary aim of this study was to investigate the relationship between UHR and lesion severity in patients who have undergone LMCA IVUS. Thus, it was thought that in patients with ICS detected in the LMCA and the decision made to perform IVUS, the UHR parameter, which can be easily measured before the IVUS procedure, can predict the severity of the lesion and prevent unnecessary IVUS procedures. There is no previous study in the literature that has investigated the relationship between UHR and lesion severity on LMCA IVUS.

MATERIALS AND METHODS

Data Collection and Laboratory Analysis

In this retrospectively planned, observational study included patients who underwent CAG at a top-level cardiac centre and were diagnosed with CAD, were determined with ICS in the LMCA as a result of CAG, and therefore underwent IVUS measurement. Non-ST segment elevation myocardial infarction (NSTEMI) and USAP (unstable angina pectoris) patients included in the study were defined as acute coronary syndrome (ACS), and other patients were defined as stable angina pectoris (SAP). The patients included were those without complications (no ulceration, dissection, or thrombus), determined with ICS and unprotected LMCA lesion. All the patients between January 2020 and June 2023 were included in the study.

The inclusion criteria for our study were defined as (i) age in the range of 20-85 years, (ii) indication for CAG with a diagnosis of SAP or ACS, and (iii) lesion determined on the LMCA as a result of CAG. Exclusion criteria from our study were defined as (i) cardiogenic shock, (ii) severe heart valve disease, (iii) active malignancy or active infection, and (iv) incomplete clinical data for uric acid or HDL-C.

A total of 205 patients who met the inclusion criteria were included in our study. The patient population was classified according to the IVUS measurements by evaluating 2 criteria. The patients were divided into 2 groups according to the healthy vessel diameter measured on IVUS, as minimal lumen area (MLA) $<6mm^2$ and $\geq 6mm^2$, and as plaque burden (PB) measured on IVUS as $<65\%$ and $\geq 65\%$. The limits defined for MLA and PB were determined according to the limits determined for IVUS in previous studies [12, 13]. During the study, all the patients were treated in the hospital according to the current guidelines. IVUS was not performed on patients presenting with ACS in the acute period. After the patients became stable, IVUS was performed. The basic demographic characteristics of the patients and comorbidities such as hypertension, diabetes mellitus, and hyperlipidemia, together with the laboratory parameters and angiographic findings were retrieved from the hospital database. From a scan of the archived records, data were also obtained for all the patients of previous ECG recordings and previous drugs used.

Routine blood tests of the patients at the time of admission were scanned from the hospital system and hemoglobin level, white blood cell level, platelet level and biochemistry values were recorded. Total cholesterol, high-density lipoprotein cholesterol and low-density lipoprotein cholesterol values were calculated from blood tests taken after an 8-12 hour overnight fast. The eGFR was calculated according to the MDRD formula (eGFR=186 x [creatinine/88.4]-1.154 x [age]- 0.203 x [0.742 if female, 1.210 if black]) [14].

Coronary Angiography

Standard coronary angiography was performed using a 5 or 6 French Judkins diagnostic catheter (Boston Scientific, MA, USA) and a femoral or radial approach. The reference vessel diameter was taken as the average of angiographically normal segments 10mm in length in the proximal and distal of the lesion. When a normal, clear segment could not be identified proximally (e.g., location of the ostial lesion), only the distal segment was included in the analysis.

IVUS Imaging and Analysis

The operators were not blinded to the angiography images. IVUS imaging was performed after the intracoronary nitroglycerine administration. The examinations were conducted using the system produced by the Cardiovascular Imaging Systems/Boston Scientific Corporation. The IVUS catheter was advanced to approximately 10mm distal of the lesion, the video recording was started and the coronary artery was imaged retrogradely up to the aorta. IVUS images were recorded by retracting at a speed of 0.5 mm/s using a motorized pull-back system. The examinations were recorded on the integral memory of the Boston device for offline analysis.

Normal coronary anatomy, plaque composition of coronary vessels, and measurements calculated using IVUS have been reported [15, 16]. The external elastic membrane (EEM) cross-sectional area is measured by following the anterior edge of the vascular adventitia. The lesion region is the cross-sectional slice with the smallest lumen and from the sections with the same lumen area, the section that included the highest plaque burden was selected. If the plaque around the catheter was compressed, the vessel lumen was assumed to be the physical size (not acoustic) of the catheter. For the reference segment, the average was taken of the visually most normal slices (the largest lumen containing the least plaque) from among the main branches 5mm proximal and distal of the lesion. For ostial lesions, the distal reference was used. IVUS measurements of the patients were recorded and the IVUS measurements were performed according to the American Cardiology College and European Cardiology Society standards [17].

Manually taken recordings were examined and the following lesion and reference measurements were performed in diastole: EEM cross-sectional areas (CSA), lumen CSA, MLA and PB ([vessel area – lumen area] /vessel area).

Statistical Analysis

All statistical analyses were performed using SPSS vn. 20 software (Statistical Package for the Social Sciences). Conformity of the data to normal distribution was assessed using the Kolmogorov–Smirnov or Shapiro–Wilk tests. Variables were reported as mean ± standard deviation (SD) or median and interquartile range values. According to the normality test result, the Independent Samples t-test or the Mann Whitney U-test were used for between group comparisons. In the analysis of categorical data, a $\chi 2$ test was used or Fisher's Exact test if any expected cell count was <5, and descriptive statistics were presented as number (n) and percentage (%). To evaluate the correlations between UHR and anatomically significant atherosclerotic parameters (plaque burden and MLA), Spearman's ρ correlation analysis was performed. ROC curve analysis was applied to determine the cut-off point and the area under the curve (AUC) of UHR for significant anatomic atherosclerosis. The cut-off values of UHR were shown as a prespecified dichotomous variable to facilitate meaningful clinical interpretation of the results. When indicated, analyses performed using UHR as a continuous variable were also reported. Univariate binary logistic regression analysis was used to investigate the relationship between significant anatomic atherosclerosis and UHR (separately for plaque burden and MLA). The effect of UHR on atherosclerosis severity was reported using the odds ratio (OR) and 95% CI. A value of $p< 0.05$ was considered statistically significant and statistical tests were two-sided.

RESULTS

The study included 205 patients who underwent CAG with a diagnosis of ACS or SAP, and were determined with an LMCA lesion on CAG, so underwent IVUS. The patients comprised 156 (76.1%) males and 49 (23.9%) females with a median (IQR) age of 61 (53-68) years. CAG was performed because of a diagnosis of ACS in 114 (55.6%) patients and SAP in 91 (44.4%). When the IVUS results were examined, the mean MLA was calculated as 6.4 (4.8-9.1) mm^2, and the plaque burden percentage as 58% (46-67%).

The correlations of anatomic atherosclerosis severity with UHR were evaluated using Spearman's ρ correlation analysis. A mild correlation was determined of both plaque burden and MLA with UHR. MLA was negatively associated with UHR, and plaque burden showed a positive correlation ($rs = -0.26$, $p=0.002$; $rs = 0.22$, $p= 0.001$,respectively). **(Figure-1)**

The demographic, clinical and laboratory data of the patients were compared in two groups according to the PB percentage of <65% or ≥ 65% in the LMCA IVUS measurements (**Table-1**). No significant difference was determined between these two groups in respect of demographic characteristics or comorbidities. When the patients were compared in terms of medication use, B-Blocker use (p:0.002) and ACE inhibitors use (p:0.022) were significantly higher in the patient group with PB<65%. When laboratory parameters were examined, it was determined that the uric acid level was significantly higher in the group with high PB (p:0.002). When the continuous variable of the UHR parameter, which shows the relationship between uric acid and HDL-C, was examined, it was determined that it was significantly higher in the group with high PB (479.5 vs. 428.6, p:0.001).

The results of the Receiver operating characteristic (ROC) analysis performed according to the MLA and PB values of the patients are shown in **Figure-2**. When the ROC analyses were applied separately to the IVUS parameters of MLA and PB with UHR, the UHR cut-off value of 450 for both parameters of anatomic atherosclerosis was determined to have similar sensitivity and the same specificity.

When UHR was examined as a categorical variable, it was determined that the number of patients with UHR ≤450 was statistically significantly higher in the low PB group (81 patients vs. 28 patients, p:0.001). No significant difference was detected in other laboratory parameters.

The demographic, clinical and laboratory data of the patients were compared as MLA <6mm^2 and MLA ≥6mm^2 according to the MLA measurement in the LMCA IVUS evaluations. (**Table-2**). No significant difference was found between the 2 patient groups in terms of demographic characteristics and comorbidities. When the patients were compared in terms of medication use, B-Blocker use (p:0.004) and ACE inhibitor use (p:0.003) were found to be significantly higher in the patient group with MLA≥6mm^2. When the laboratory parameters were examined, the UHR variable was determined to be statistically significantly higher in the low MLA group (476.8 vs. 414.9, p<0.001). The UHR variable was examined as a categorical variable and it was determined that the number of patients with UHR ≤450 was significantly higher in the high MLA group (74 patients vs. 35 patients, p<0.001). It was determined that the PB percentage was statistically significantly higher in the low MLA group (p<0.001).

The angiographic characteristics and IVUS parameters of the patients were compared according to the UHR levels as ≤450 and >450 (**Table-3**). It was observed that the number of critical vessels determined angiographically was significantly higher in the high UHR group (p:0.039). When the CAG results were compared, the number of patients for whom CABG was decided was significantly higher in the high UHR group (p:0.017). In the IVUS parameters, it was determined that the lumen volume was significantly lower in the group with high UHR (p:0.012). MLA values were determined to be significantly low in the high UHR group (6.7 mm^2 vs 8.2 mm^2, p:0.030). When the MLA parameter was examined as a categorical variable, the number of patients with MLA <6mm^2 was significantly greater in the high UHR group (57 patients vs.35 patients, p<0.001). The PB percentage was determined to be significantly higher in the high UHR group (p.0.002). When the PB percentage was examined as a categorical variable, the number of patients with PB >65% was significantly greater in the high UHR group (p: 0.001).

DISCUSSION

The results of this study demonstrated that significantly higher UHR values were determined in patients determined with a critical lesion according to the two separate parameters of MLA and plaque burden in the IVUS measurements of patients determined with ICS on LMCA. The UHR showed a negative correlation with MLA, showing lesion severity in the LMCA IVUS measurements, and a positive correlation with PB. The UHR, a novel biomarker combining UA and HDL-C, showed excellent diagnostic capacity for anatomically significant stenosis in patients with ICS detected in the LMCA. To our knowledge, this is the first study in the literature to investigate the anatomical relationship between UHR and lesion severity in patients with ICS detected in the LMCA.

Despite advances in the field of interventional cardiology, correct evaluation of ICS in the catheterisation laboratory and the decision for PCI are still a great challenge [18]. This problem has been resolved by determining the plaque burden and lesion severity with MLA limits through comparisons of IVUS measurements in LMCA lesions with distal and proximal vessel diameters. An MLA value <6mm^2 and plaque burden ≥65% shows anatomically critical LMCA stenosis [12, 13]. However, IVUS devices are not widely used in diagnosis and treatment in interventional cardiology in clinical practice due to extra operating time, high cost, and the need for an experienced operator [19]. Therefore, there seems to be a need for a biomarker that is reliable in terms of showing lesion severity in ICS lesions. Some biomarkers have been investigated in previous studies to determine the severity of lesions in patients diagnosed with ICS. However, those studies have focussed more on the functional significance of the stricture. Erdoğan M. et al. suggested that the systemic immune inflammation index, calculated as the number of neutrophils*platelets/lymphocytes, could predict fractional flow reserve (FFR) with high sensitivity and specificity [5]. Other studies have also shown that the UA value and modification of some lipid biomarkers could predict the FFR value [6, 20]. In a recent study by Fanqi Li et al., a significant relationship was found between UHR and FFR severity in patients with ICS [21]. All of these studies concentrated on the functional significance of the stricture and were conducted with the FFR test.

IVUS imaging is the current gold standard method to evaluate LMCA lesions [22]. In intermediate LMCA lesions, the FFR test often causes incorrect classification [23]. In addition, because of the high cost, reimbursement for IVUS imaging is only received for LMCA lesions in Türkiye. Therefore, only patients with LMCA lesions were included in this study.

In the current study population, uric acid values were determined to be significantly higher in the patient group with high plaque burden. In a previous study by Ando K. et al. on this subject, a significant relationship was found between high uric acid and high lipid volume and plaque burden on IVUS [24]. Unlike the current study, that study included IVUS measurements for vessels other than the LMCA, and the effect of uric acid on plaque

burden in particular was shown to be independent of gender. In this study, it was determined that uric acid levels were significantly higher in the patient group with <6mm^2. As there was seen to be a relationship between uric acid levels and a high plaque burden, it was expected that MLA would be low in cases with high uric acid levels.

When the UHR parameter was examined as a continuous variable, a statistically significant relationship was determined between the UHR variable and high plaque burden and low MLA values. The HDL-C parameter alone, independently of uric acid, was not found to have a significant effect on these measurements. This result could be associated with being lower in the group with high plaque burden and low MLA value, even if the HDL-C values were not significant. This can explain the significant effect of the HDL-C value on plaque burden and MLA compared to uric acid.

When ROC curve analyses were performed for MLA and plaque burden separately with the UHR parameter, the UHR cut-off value was found to be 450 for both curves. This demonstrated that a value of 450 can be defined as the limit for the UHR parameter. The patient population was then evaluated as subgroups above and below the UHR value of 450. It was determined that the number of patients with MLA <6mm^2 and plaque burden ≥65% was significantly higher in the patient group with high UHR value. Recently at a FFR related study, the UHR cut-off value was determined to be 310.8 [21]. This determined cutoff value was a much lower cutoff value than in our current study, and this difference can be attributed to the measurement methods. The above-mentioned study evaluated the stricture functionally using an FFR device, whereas anatomic stricture evaluation was performed in the current study using IVUS.

In our study, the rate of B-blocker and ACE inhibitor use was significantly lower in the patient group with MLA <6 mm^2 and the patient group with plaque burden ≥65%. Previous studies in literature have shown that B-blocker use slows the progression of coronary atherosclerosis [25]. It has also been shown that ACE inhibitor use significantly reduces coronary atherosclerosis [26]. Consistent with the findings of those studies, the use of B-blockers and ACE inhibitors was determined to be high in the current study patients with low plaque burden and high MLA.

From these results, it was seen that the UHR parameter can predict vessel stricture, consistent with MLA and plaque burden showing critical vascular narrowing on IVUS measurements in patients with ICS LMCA lesion. Thus it was shown that UHR can predict values of MLA <6mm^2 and plaque burden ≥65%, which are accepted as critical in IVUS measurements for LMCA.

Study Limitations

There were some limitations to this study to be considered, primarily the single-centre, retrospective design and limited sample size. Only patients with LMCA lesion were included in the study, because IVUS is the gold standard method in LMCA lesions and in Türkiye, reimbursement for use of the IVUS device is only made for LMCA lesions. A further limitation was that the rate of statin use was high in the patient population and this could have affected the HDL-C value in the UHR calculation. However, as there was no significant difference in the rate of statin use between those with and without critical lesions, statin use was not considered to have affected the results obtained.

CONCLUSION

The results of this study suggested that there was a relationship between the UHR parameter and MLA <6mm^2 and plaque burden ≥65%, which are independently evaluated as critical in IVUS, and this could predict anatomically significant lesions in patients with a moderate degree of LMCA stricture.

REFERENCES

1. GBD 2013 Mortality and Causes of Death Collaborators (2015). Global, regional, and national age-sex specific all-cause and cause-specific mortality for 240 causes of death, 1990-2013: a systematic analysis for the Global Burden of Disease Study 2013. Lancet 385(9963):117-171.
2. Patil CV, Beyar R (2000) Intermediate coronary artery stenosis: evidence-based decisions in interventions to avoid the oculostenotic reflex. Int J Cardiovasc Intervent 3(4):195-206.
3. Patel MR, Peterson ED, Dai D, Brennan JM, Redberg RF, Anderson HV, et al. (2010) Low diagnostic yield of elective coronary angiography. N Engl J Med 11;362(10):886-95.
4. Sibbald M, Chan W, Daly P, Horlick E, Ing D, Ivanov J, et al. (2014) Long-term outcome of unprotected left main stenting: a Canadian tertiary care experience. Can J Cardiol 30(11):1407-14.
5. Erdoğan M, Erdöl MA, Öztürk S, Durmaz T (2020) Systemic immune-inflammation index is a novel marker to predict functionally significant coronary artery stenosis. Biomark Med 14(16):1553-1561.
6. Topal S, Sezenöz B, Candemir M, Açikgöz E, Açikgöz SK, Boyaci NB (2019) An old friend: uric acid and its association with fractional flow reserve. Turk J Med Sci 49(6):1614-1619.
7. Saito Y, Tanaka A, Node K, Kobayashi Y (2021) Uric acid and cardiovascular disease: A clinical review. J Cardiol 78(1):51-57.
8. Kosmas CE, Christodoulidis G, Cheng JW, Vittorio TJ, Lerakis S (2014) High-density lipoprotein functionality in coronary artery disease. Am J Med Sci 347(6):504-8.

9. Aktas G, Khalid A, Kurtkulagi O, Duman TT, Bilgin S, Kahveci G, et al. (2022) Poorly controlled hypertension is associated with elevated serum uric acid to HDL-cholesterol ratio: a cross-sectional cohort study. Postgrad Med 134(3):297-302.
10. Kosekli MA, Kurtkulagii O, Kahveci G, Duman TT, Tel BMA, Bilgin S, et al. (2021) The association between serum uric acid to high density lipoprotein-cholesterol ratio and non-alcoholic fatty liver disease: the abund study. Rev Assoc Med Bras 67(4):549-554.
11. Liu R, Peng Y, Wu H, Diao X, Ye H, Huang X, et al. (2021) Uric acid to high-density lipoprotein cholesterol ratio predicts cardiovascular mortality in patients on peritoneal dialysis. Nutr Metab Cardiovasc Dis 31(2):561-569.
12. Stone GW, Maehara A, Ali ZA, Held C, Matsumura M, Kjøller-Hansen L, et al. PROSPECT ABSORB Investigators. Percutaneous Coronary Intervention for Vulnerable Coronary Atherosclerotic Plaque. J Am Coll Cardiol. 2020 Nov 17;76(20):2289-2301. doi: 10.1016/j.jacc.2020.09.547. Epub 2020 Oct 15. PMID: 33069847.
13. de la Torre Hernandez JM, Hernández Hernandez F, Alfonso F, Rumoroso JR, Lopez-Palop R, Sadaba M, et al. LITRO Study Group (Spanish Working Group on Interventional Cardiology). Prospective application of pre-defined intravascular ultrasound criteria for assessment of intermediate left main coronary artery lesions results from the multicenter LITRO study. J Am Coll Cardiol. 2011 Jul 19;58(4):351-8. doi: 10.1016/j.jacc.2011.02.064. PMID: 21757111.
14. Levey AS, Bosch JP, Lewis JB, Greene T, Rogers N, Roth D (1999) A more accurate method to estimate glomerular filtration rate from serum creatinine: a new prediction equation. Modification of Diet in Renal Disease Study Group. Ann Intern Med 130(6):461-70.
15. Potkin BN, Bartorelli AL, Gessert JM, Neville RF, Almagor Y, Roberts WC, et al. (1990) Coronary artery imaging with intravascular high-frequency ultrasound. Circulation 81(5):1575-85.
16. Nissen SE, Grines CL, Gurley JC, Sublett K, Haynie D, Diaz C, et al. (1990) Application of a new phased-array ultrasound imaging catheter in the assessment of vascular dimensions. In vivo comparison to cineangiography. Circulation 81(2):660-6.
17. Mintz GS, Nissen SE, Anderson WD, Bailey SR, Erbel R, Fitzgerald PJ, et al. (2001) American College of Cardiology Clinical Expert Consensus Document on Standards for Acquisition, Measurement and Reporting of Intravascular Ultrasound Studies (IVUS). A report of the American College of Cardiology Task Force on Clinical Expert Consensus Documents. J Am Coll Cardiol 37(5):1478-92.
18. Tobis J, Azarbal B, Slavin L (2007) Assessment of intermediate severity coronary lesions in the catheterization laboratory. J Am Coll Cardiol 49(8):839-48.
19. Koskinas KC, Nakamura M, Räber L, Colleran R, Kadota K, Capodanno D, et al. (2018) Current use of intracoronary imaging in interventional practice - Results of a European Association of Percutaneous Cardiovascular Interventions (EAPCI) and Japanese Association of Cardiovascular Interventions and Therapeutics (CVIT) Clinical Practice Survey. EuroIntervention 14(4):e475-e484.
20. Muller O, Ntalianis A, Wijns W, Delrue L, Dierickx K, Auer R, et al. (2013) Association of biomarkers of lipid modification with functional and morphological indices of coronary stenosis severity in stable coronary artery disease. J Cardiovasc Transl Res 6(4):536-44.
21. Li F, Zhao D, Li Q, Lin X, Sun H, Fan Q (2022) Uric Acid to High-Density Lipoprotein Cholesterol Ratio is a Novel Marker to Predict Functionally Significant Coronary Artery Stenosis. J Interv Cardiol 2022:9057832.
22. Noguchi M, Gkargkoulas F, Matsumura M, Kotinkaduwa LN, Hu X, Usui E, et al. (2022) Impact of Nonobstructive Left Main Coronary Artery Atherosclerosis on Long-Term Mortality. JACC Cardiovasc Interv 15(21):2206-2217.
23. Toth G, Hamilos M, Pyxaras S, Mangiacapra F, Nelis O, De Vroey F, et al. (2014) Evolving concepts of angiogram: fractional flow reserve discordances in 4000 coronary stenoses. Eur Heart J 35(40):2831-8.
24. Ando K, Takahashi H, Watanabe T, Daidoji H, Otaki Y, Nishiyama S, et al. (2016) Impact of Serum Uric Acid Levels on Coronary Plaque Stability Evaluated Using Integrated Backscatter Intravascular Ultrasound in Patients with Coronary Artery Disease. J Atheroscler Thromb 23(8):932-9.
25. Sipahi I, Tuzcu EM, Wolski KE, Nicholls SJ, Schoenhagen P, Hu B, et al. (2007) Beta-blockers and progression of coronary atherosclerosis: pooled analysis of 4 intravascular ultrasonography trials. Ann Intern Med 147(1):10-8.
26. Borghi C, Levy BI (2022) Synergistic actions between angiotensin-converting enzyme inhibitors and statins in atherosclerosis. Nutr Metab Cardiovasc Dis 32(4):815-826.

TABLES

Table 1: The baseline characteristics and laboratory investigations of all patients according to LMCA plaque burden

	All patients (n=205)	Plaque Burden $\geq$%65 (n=74)	Plaque Burden <65 (n=131)	p-value
**Demographic characteristics**				
Age, y	61 (53-68)	60 (55-71)	61 (53-68)	0.186
Male sex, %	156 (76.1)	55 (74.3)	101 (77.1)	0.734
Body mass index, kg/m^2	26.8 (24.7-29)	26.2 (24.5-29.3)	26.8 (24.8-28.9)	0.605
**Comorbidites**				
Hypertension, %	129 (62.9)	45 (60.8)	84 (64.1)	0.654
Diabetes mellitus, %	48 (23.4)	19 (25.7)	29 (22.1)	0.608
Hyperlipidemia, %	60 (29.3)	27 (36.5)	33 (25.2)	0.110
Smoking, %	132 (64.4)	47 (63.5)	85 (64.9)	0.880
Chronic kidney disease, %	31 (15.1)	12 (16.2)	19 (14.5)	0.840
**Medications**				
Acetylsalicylic Acid Use, %	204 (99.5)	74 (100)	130 (99.2)	1.000
P2Y12 Inh Use, % — Clopidogrel	103 (50.2)	40 (54.1)	63 (48.1)	0.468
Ticagrelor	44 (21.5)	22 (29.7)	22 (16.8)	
Prasugrel	16 (7.8)	6 (8.1)	10 (7.6)	
B-Blocker Use, %	191 (93.2)	60 (82.4)	131 (100)	0.002
ACE Inh Use, %	175 (85.4)	52(81.1)	123(93.9)	0.022
Statin Use, %	200 (97.6)	73 (98.6)	127 (96.9)	0.656
OAD Use, %	50 (24.4)	24 (32.4)	26 (19.8)	0.062
Allopurinol Use, %	16 (7.8)	6 (8.1)	10 (7.6)	0.716
**Clinical Presentation**				
Acute Coronary Syndrome, %	114 (55.6)	47 (63.5)	67 (51.1)	0.094
Stable Angina Pectoris, %	91 (44.4)	27 (36.5)	64 (48.9)	
**Laboratory assessment**				
Hemoglobin, g/dl	13.3 (12.1-14.5)	12.8 (11.7-14.3)	13.6 (12.2-14.6)	0.085
eGFR, ml/min/1.73 m^2	90.5 (70-101.6)	92.8 (70-101.5)	90.1 (69.8-102.1)	0.904
Total cholesterol, mmol/L	4.37 (3.74-5.3)	4.25 (3.43-5.30)	4.52 (3.87-5.36)	0.086
Triglycerides, mmol/L	1.56 (1.01-2.41)	1.41 (0.97-2.16)	1.60 (1.06-2.53)	0.279
LDL cholesterol, mmol/L	2.39 (1.78-3.33)	2.31 (1.57-3.07)	2.44 (1.84-3.48)	0.123
HDL cholesterol, mmol/L	1.07 (0.94-1.25)	1.07 (0.91-1.25)	1.08 (0.94-1.26)	0.456
Albumin, g/L	42.1 (39.1-44.5)	41.7 (38.4-44.3)	42.1 (39.1-44.6)	0.115
HbA1c, %	5.78 (5.5-6.4)	5.89 (5.6-6.5)	5.69 (5.4-6.4)	0.430
Uric asid, µmol/L	477.4 (415.5-548.1)	503.9 (459.7-565.8)	468.5 (406.6-539.2)	0.002
UHR, continuous variable	446.3 (366-518.1)	479.5 (400.4-570)	428.6 (357-494.3)	0.001
UHR ≤450, categorical variable	109 (53,2)	28 (37.8)	81 (61.8)	0.001
IVUS LMCA MLA, mm^2	6.4 (4.8-9.1)	4.9 (3.5-5.8)	8.7 (6.4-10.9)	<0.001
IVUS LMCA plaque burden, %	58 (46-67)	69 (66-72)	48 (41-56)	<0.001

Table 2: The baseline characteristics and laboratory investigations of all patients according to LMCA minimum lumen area

		All patients (n=205)	MLA <6mm² (n=92)	MLA ≥6mm² (n=113)	p-value
**Demographic characteristics**					
Age, y		61 (53-68)	60 (55-70)	61 (52-68)	0.158
Male sex, %		156 (76.1)	72 (78.3)	84 (74.3)	0.512
Body mass index, kg/m²		26.8 (24.7-29)	26 (24.2-28.6)	27 (24.9-29)	0.097
**Comorbidites**					
Hypertension, %		129 (62.9)	58 (63)	71 (62.8)	1.000
Diabetes mellitus, %		48 (23.4)	25 (27.2)	23 (20.4)	0.320
Hyperlipidemia, %		60 (29.3)	31 (33.7)	29 (25.7)	0.221
Smoking, %		132 (64.4)	56 (60.9)	76 (67.3)	0.380
Chronic kidney disease, %		31 (15.1)	14 (15.2)	17 (15)	1.000
**Medications**					
Acetylsalicylic Acid Use, %		204 (99.5)	92 (100)	112 (99.1)	1.000
P2Y12 Inh Use, %	Clopidogrel	103 (50.2)	50 (54.3)	53 (46.9)	0.327
	Ticagrelor	44 (21.5)	27 (29.3)	17 (15)	
	Prasugrel	16 (7.8)	7 (7.6)	9 (8)	
B-Blocker Use, %		191 (93.2)	81 (85.9)	110 (97.3)	0.004
ACE Inh Use, %		175 (85.4)	70 (79.3)	105 (93)	0.003
Statin Use, %		200 (97.6)	91 (98.9)	109 (96.5)	0.382
OAD Use, %		50 (24.4)	27 (29.3)	23 (20.4)	0.145
Allopurinol Use, %		16 (7.8)	7 (7.6)	9 (8)	0.738
**Clinical Presentation**					
Acute Coronary Syndrome, %		114 (55.6)	60 (65.2)	54 (47.8)	0.074
Stable Angina Pectoris, %		91 (44.4)	32 (34.8)	59 (52.2)	
**Laboratory assessment**					
Hemoglobin, g/dl		13.3 (12.1-14.5)	13.1 (11.9-14.4)	13.6 (12.2-14.5)	0.630
eGFR, ml/min/1.73 m²		90.5 (70-101.6)	92.5 (70-101.5)	88.5 (70-101.6)	0.995
Total cholesterol, mmol/L		4.37 (3.74-5.3)	4.26 (3.44-5.31)	4.53 (3.88-5.37)	0.084
Triglycerides, mmol/L		1.56 (1.01-2.41)	1.41 (0.97-2.16)	1.62 (1.08-2.55)	0.259
LDL cholesterol, mmol/L		2.39 (1.78-3.33)	2.29 (1.55-3.08)	2.47 (1.84-3.51)	0.096
HDL cholesterol, mmol/L		1.07 (0.94-1.25)	1.06 (0.91-1.24)	1.08 (0.96-1.28)	0.107
HbA1c, %		5.78 (5.5-6.4)	5.88 (5.5-6.4)	5.69 (5.3-6.4)	0.353
Uric asid, µmol/L		477.4 (415.5-548.1)	503.9 (446.4-565.8)	468.5 (406.6-521.6)	0.012
UHR, continuous variable		446.3 (366-518.1)	476.8 (405.5-560)	414.9 (349.4-489)	<0.001
UHR ≤450, categorical variable		109 (53.2)	35 (38)	74 (65.5)	<0.001
IVUS LMCA MLA, mm²		6.4 (4.8-9.1)	4.8 (3.6-5.1)	8.9 (6.7-11.6)	<0.001
IVUS LMCA plaque burden, %		58 (46-67)	66.5 (64-71)	47 (40-55)	<0.001

Table 3: Angiographic and procedural status of patients according to UHR

Angiographic parameters		All patients (n=205)	UHR ≤ 450 (n=109)	UHR > 450 (n=96)	p-value
**Procedural data**					
SYNTAX score		11 ± 10	10 ± 10	12 ± 9	0.061
Critical number of vessels, %	0	46 (22.4)	33 (30.3)	13 (13.5)	0.039
	1	64 (31.2)	33 (30.3)	31 (32.3)	
	2	59 (28.8)	26 (23.9)	33 (34.4)	
	3	35 (17.1)	16 (14.7)	19 (19.8)	
	4	1 (0.5)	1 (0.9)	0	
CAG result	Medical follow up	80 (39)	51 (46.8)	29 (30.2)	0.017
	PCI	73 (35.6)	38 (34.9)	35 (36.5)	
	CABG	52 (25.4)	20 (18.3)	32 (33.3)	
**IVUS-based volume parameters in LMCA**					
EEM volume, mm^3		148.9 ± 47	151.3 ± 45.2	146.2 ± 49	0.434
Lumen volume, mm^3		66.5 ± 35.7	72.4 ± 36.9	59.8 ± 33.4	0.012
Plaque volume, mm^3		82.4 ± 32.1	78.9 ± 28.8	86.4 ± 35.1	0.095
MLA, mm^2		7.4 ± 3.8	8.2 ± 3.9	6.7 ± 3.5	0.030
MLA< 6 mm^2		92 (44.9)	35 (32.1)	57 (59.4)	<0.001
Plaque burden, %		55.6 ± 14.5	52.7 ±14.2	59 ± 14.2	0.002
Plaque burden ≥ 65%		74 (36.1)	28 (25.7)	46 (47.9)	0.001

FIGURE LEGENDS

Figure-1: Correlations between UHR and atherosclerotic severity variables

Figure-2: ROC analysis of UHR according to MLA and PB

makalE

ORIGINALITY REPORT

19%	15%	18%	0%
SIMILARITY INDEX	INTERNET SOURCES	PUBLICATIONS	STUDENT PAPERS

PRIMARY SOURCES

1	Ömer Furkan Demir, Fatih Koca. "The relationship between triglyceride/high-density lipoprotein cholesterol ratio and the severity of coronary artery disease in patients presenting with acute coronary syndrome", Coronary Artery Disease, 2024<br>Publication	4%
2	Ömer Furkan Demir, Nur Özer Şensoy, Esra Akpınar, Günseli Demir. "The stress hyperglycemic ratio can predict the no-reflow phenomenon following saphenous vein graft intervention in patients with acute coronary syndrome", Acta Diabetologica, 2023<br>Publication	3%
3	circ.ahajournals.org<br>Internet Source	2%
4	www.hindawi.com<br>Internet Source	2%
5	dergipark.org.tr<br>Internet Source	1%

6	gcris.pau.edu.tr<br>Internet Source	1%
7	medscidiscovery.com<br>Internet Source	1%
8	www.biomedcentral.com<br>Internet Source	1%
9	hydra.hull.ac.uk<br>Internet Source	<1%
10	research.vumc.nl<br>Internet Source	<1%
11	Abdullah Kadir Dolu, Filiz Akyıldız Akçay, Murat Atalay, Mustafa Karaca. "Systemic Immune-Inflammation Index as a Predictor of Left Atrial Thrombosis in Nonvalvular Atrial Fibrillation", The Journal of Tehran University Heart Center, 2023<br>Publication	<1%
12	japer.in<br>Internet Source	<1%
13	"PanVascular Medicine", Springer Nature, 2015<br>Publication	<1%
14	arsiv.tkd.org.tr<br>Internet Source	<1%
15	link.springer.com<br>Internet Source	

		<1%
16	bmcpublichealth.biomedcentral.com<br>Internet Source	<1%
17	www.jstage.jst.go.jp<br>Internet Source	<1%
18	www.revistanefrologia.com<br>Internet Source	<1%
19	Abizaid, A.S.. "One-year follow-up after intravascular ultrasound assessment of moderate left main coronary artery disease in patients with ambiguous angiograms", Journal of the American College of Cardiology, 199909<br>Publication	<1%
20	Jason Nogic, Hamish Prosser, Joseph O'Brien, Udit Thakur, Kean Soon, George Proimos, Adam J. Brown. "The assessment of intermediate coronary lesions using intracoronary imaging", Cardiovascular Diagnosis and Therapy, 2020<br>Publication	<1%
21	www.ecco-ibd.eu<br>Internet Source	<1%
22	ifcardio.org<br>Internet Source	<1%

23	www.dovepress.com<br>Internet Source	<1 %

Exclude quotes	On	Exclude matches	< 14 words
Exclude bibliography	On		
